# Rapid and Efficient Isolation of High-Quality Small RNAs from Recalcitrant Plant Species Rich in Polyphenols and Polysaccharides

**DOI:** 10.1371/journal.pone.0095687

**Published:** 2014-05-01

**Authors:** Jun Peng, Zihao Xia, Ling Chen, Minjing Shi, Jinji Pu, Jianrong Guo, Zaifeng Fan

**Affiliations:** 1 State Key Laboratory of Agro-biotechnology and Ministry of Agriculture Key Laboratory for Plant Pathology, China Agricultural University, Beijing, China; 2 Ministry of Agriculture Key Laboratory of Integrated Pest Management on Tropical Crops, Environmental and Plant Protection Institute, Chinese Academy of Tropical Agricultural Sciences, Haikou, Hainan, China; 3 Ministry of Agriculture Key Laboratory for Rubber Biology, Rubber Research Institute, Chinese Academy of Tropical Agricultural Sciences, Danzhou, Hainan, China; TGen, United States of America

## Abstract

Small RNAs, including microRNAs (miRNAs) and small interfering RNAs (siRNAs), are important regulators of plant development and gene expression. The acquisition of high-quality small RNAs is the first step in the study of its expression and function analysis, yet the extraction method of small RNAs in recalcitrant plant tissues with various secondary metabolites is not well established, especially for tropical and subtropical plant species rich in polysaccharides and polyphenols. Here, we developed a simple and efficient method for high quality small RNAs extraction from recalcitrant plant species. Prior to RNA isolation, a precursory step with a CTAB-PVPP buffer system could efficiently remove compounds and secondary metabolites interfering with RNAs from homogenized lysates. Then, total RNAs were extracted by Trizol reagents followed by a differential precipitation of high-molecular-weight (HMW) RNAs using polyethylene glycol (PEG) 8000. Finally, small RNAs could be easily recovered from supernatant by ethanol precipitation without extra elimination steps. The isolated small RNAs from papaya showed high quality through a clear background on gel and a distinct northern blotting signal with miR159a probe, compared with other published protocols. Additionally, the small RNAs extracted from papaya were successfully used for validation of both predicted miRNAs and the putative conserved tasiARFs. Furthermore, the extraction method described here was also tested with several other subtropical and tropical plant tissues. The purity of the isolated small RNAs was sufficient for such applications as end-point stem-loop RT-PCR and northern blotting analysis, respectively. The simple and feasible extraction method reported here is expected to have excellent potential for isolation of small RNAs from recalcitrant plant tissues rich in polyphenols and polysaccharides.

## Introduction

MicroRNAs (miRNAs) and small interfering RNAs (siRNAs) are important regulators of plant development and gene expression [1]. The major differences between these two categories lie in their genomic origin and biogenesis. In plants, miRNA primary transcripts arise from intergenic regions via the action of RNA polymerase II [2]. MiRNAs are processed from their precursors by RNase III enzyme DICER-LIKE (DCL), which digests the imperfectly complementary hairpin structure of precursors into miRNA:miRNA* duplexes [3]. The mature miRNAs of the duplexes combine with protein factors to form RNA-induced silencing complexes (RISCs). Subsequently, miRNAs guide the RISCs to target messenger RNA (mRNA) molecules, where they regulate mRNAs primarily at the post-transcriptional level in plants by directing mRNA cleavage or translation inhibition via the endoribonuclease activity of the Argonaute (AGO) protein [1], [4], [5].

*Trans*-acting small interfering RNAs (tasiRNAs) belong to a specialized class of small RNAs that originate from *TAS* gene-derived transcripts which are cleaved at one or two miRNA target site, and act *in trans* to regulate mRNAs at the post-transcriptional level [6]–[10]. The *TAS3* family is highly conserved in land plants and distinguished from other *TAS* loci by the dual miR390 complementary sites flanking the tasiRNA region and its dependence on AGO7 [7], [9]. All the known *TAS3* genes contain single or tandem putative tasiARFs in 21-nt phasing registers [11]. In Arabidopsis, two near-identical conserved 21-nt tasiARFs, tasiR2141 and tasiR2142, co-align with the phases 5′D7 [+] and 5′D8 [+], which target Auxin Response Factor (ARF) transcripts [9], [10]. Conserved tasiARFs have been proved to influence leaf morphology, leaf polarity, developmental timing, and patterning by targeting *ARF3* and *ARF4* [9], [12]–[14].

Many subtropical and tropical plants are economically important crops, such as rubber tree (*Hevea brasiliensis*), banana and plantain (*Musa* spp.) and the papaya (*Carica papaya* L.). As lack of genome sequences, the identification and functional study of small RNAs are poorly understood in subtropical and tropical plants. Moreover, these recalcitrant plant tissues contain rich polysaccharides, polyphenols, latex, tannins, pigments and other metabolites, which could bind or co-precipitate with RNAs, thereby increasing the difficulty of sample preparation and impeding the extraction of high quality small RNAs for subsequent identification and molecular analysis [15]–[17]. Indeed, the present of polyphenols together with high polysaccharide content makes the isolation of high quality nucleic acids problematic. It is necessary, therefore, to develop an efficient protocol for the isolation of high-quality small RNAs from recalcitrant plant species rich in polysaccharides and polyphenols for downstream molecular analysis.

Several methods have been used to isolate small RNAs from various plant tissues, and these protocols generally involved two major steps: start with total RNA extraction, and then isolation or separation of small RNAs [18]–[23]. And now, some improved methods have been used to isolate small RNAs from different plant species. The plant tissues were firstly dealt with either CTAB (cetyl trimethylammonium bromide) or LiCl (lithium chloride) extraction buffer followed by LiCl or polyethylene glycol (PEG) precipitation of high-molecular RNAs, and then small RNAs were obtained through isopropanol or ethanol precipitation without total RNA precipitation steps, which increases yield in a reduced number of extraction steps, and provides a possibility for rapidly isolating small RNAs from some plant materials [24], [25]. However, these protocols failed to yield high quality small RNAs for above-mentioned tropical plant tissues rich in polysaccharides, polyphenolics and secondary metabolites, and thus the need was ultimately raised to explore effective small RNAs extraction protocols for certain recalcitrant plant materials. The modification of existing protocols or the development of new procedures is required for the isolation of large amounts of small RNAs from these recalcitrant plant materials [15], [17].

High-quality small RNAs extracted from these plant species are crucial for the subsequent detection and molecular analysis. Some total RNA extraction methods from plant species rich in polyphenols or polysaccharides have been previously reported, which offered references on improving small RNAs isolation from the same plant materials. Methods involving CTAB have been successfully used to extract total RNA from a wide range of polysaccharide- and polyphenol-rich plant tissues [15], [26]–[33]. The phenolic compounds are bound to insoluble PVPP and then eliminated by centrifuge or precipitation [34], [35]. In addition, guanidinium thiocyanate was the most widely used RNA extraction reagent for inhibiting RNase activity [18], [19], [35]–[37]. Among these successful precedents, compounds that may interfere with RNA were eliminated by precipitation as a precursory step to RNA extraction. Accordingly, the effective removal of interfering substances prior to small RNAs extraction is a prerequisite factor to ensure the quality of the resulting small RNAs.

Therefore, by modifying and combining different techniques that address the aforementioned problems simultaneously, we have developed a protocol to rapidly and effectively isolate high-quality small RNAs from plant species rich in secondary metabolites. In the modified protocol described here, a precursory step prior to RNA extraction was developed, which could effectively eliminate polyphenolic compounds and polysaccharides interfering with RNA from homogenized lysates. After that, total RNAs were then extracted by Trizol reagent and followed by differential precipitated of high-molecular-weight (HMW) RNAs with PEG 8000 from supernatant in a low salt concentration. Finally, small RNAs could be easily recovered by ethanol precipitation from supernatant without any additional elimination steps. The modified protocol directly yielded high-quality small RNAs in sufficient quantities from supernatant without the total RNA precipitation step.

Using the newly established extraction method, small RNAs isolated from papaya leaves were successfully used for experimental validation of two miRNAs, i.e. miR162a and miR403, and putative conserved tasiARFs by northern blotting analysis. The quality of isolated small RNAs was tested by other recalcitrant plant speices. The method reported here is expected to have excellent potential for small RNAs isolation from recalcitrant plant tissues rich in polysaccharides and polyphenols.

## Materials and Methods

There are no specific permissions required for these sampling locations of this study, and don’t need to provide details on why this is the case. Also, we did not require ethical approval to conduct this study as we did not handle or collect animals considered in any animal welfare regulations and no endangered or protected species were involved in the samplings or the experiments.

### Plant Materials

Leaves of different tropical and subtropical plant species, including papaya, banana, guava, citrus, pineapple, longan, lychee, rubber and cactus, were collected from tropical fruit germplasm repository in Institute of Tropical Crops and Genetic Resources, Chinese Academy of Tropical Science (CATAS), Danzhou, Hainan Island. After collection, all the samples were immediately frozen in liquid nitrogen and stored at −80°C.

### Solutions and Reagents

#### CTAB buffer

2% (w/v) CTAB, 4 M of guanidinium thiocyanate, 100 mM of Tris-HC1 (pH 8.5), 25 mM EDTA, 2 M of NaCl, 2% (w/v) PVPP, and 2% (v/v) β-mercaptoethanol (added just before use).

#### Precipitation buffer

PEG 8000 (Polyethylene glycol 8000): 20% (w/v) PEG 8000 (Amresco Inc, USA), 1 M of NaCl.

LiCl: 4 M of LiCl.

#### EDC fixation solution (12 ml, pH 8.0)

122.5 µl of 12.5 M 1-methylimidazole, 0.373 g of 1-ethyl-3-(3-dimethyl aminopropyl) carbodiimide (EDC) (Sigma, USA).

#### Hybridization buffer

5×SSC, 20 mM of Na_2_HPO_4_ (pH 7.2), 7% SDS, 3×Denhardt’s solution (Amresco Inc, USA).

### Extraction Protocol

#### Step 1. Pre-treatment prior to RNA isolation

Before extraction, mortars and pestles were baked for 6 h at 180°C, while pipette tips and centrifuge tubes were all treated with 0.1% DEPC water and autoclaved. For fresh tissues, 2–3 g of samples were ground to a fine powder with a mortar and pestle in liquid nitrogen. The frozen powder was subsequently transferred into a 50-ml centrifuge tube containing 20 ml of CTAB-PVPP lysis buffer, homogenized by vortexing for at least 30 s. The homogenate was kept at room temperature for 3–5 min, and followed by centrifugation at 12,000 rpm (17,418×*g*) (rotor JA-25.50, Avanti J-26 XP, Beckman Coulter, Inc., USA) for 5 min at 4°C.

#### Step 2. Trizol reagent extraction

After centrifugation, the upper aqueous phase was carefully transferred to a clean 50-ml centrifuge tube. An equal volume of Trizol reagent (Tiangen, Beijing) was added, thoroughly mixed by vortexing, and the mixture was placed at room temperature for 5–10 min. And then, 4 ml chloroform-isoamyl alcohol (24∶1, v/v) were added, vortexed vigorously and centrifuged at 12,000 rpm for 10 min at 4°C.

#### Step 3. Separation of HMW RNAs

The resulting upper aqueous phase was transferred into another clean 50-ml centrifuge tube. Then, an equal volume of pre-heated PEG8000 precipitation buffer were added and incubated at 65°C for 15 min, kept at room temperature for 5–10 min, and chilled on ice immediately for 30–45 min to precipitate the HMW RNAs, or precipitate the HMW RNAs using 4 M of LiCl overnight. Following centrifugation as described above, the supernatant was collected for the enrichment of LMW RNAs.

#### Step 4. Enrichment of LMW RNAs

The aqueous phase was recovered after centrifugation and transferred into a new 50-ml centrifuge tube. LMW RNAs were precipitated with 1/10 volume of 3 M sodium acetate (pH 5.2) and 2.5 volume of pre-cooled absolute ethanol at −20°C overnight. The pellet was collected by centrifugation at 12,000 rpm for 20 min at 4°C, then rinsed with 80% ice-cold ethanol and collected by centrifugation at 12,000 rpm for 5 min at 4°C. The air-dried pellet was re-suspended in an appropriate volume of sterile DEPC-treated double-distilled water and stored at −80°C.

### Analysis of Small RNAs in Polyacrylamide Gel Electrophoresis

An equal volume of loading buffer (ABI, Cat. AM8547) was added to each small RNA samples, and the mixture was incubated for 5 min at 95°C, and then chilled on ice immediately. All RNA samples were separated by electrophoresis using 17% polyacrylamide (19∶1) gel cast in 7 M urea and buffered with 0.5×TBE, using an SE600 standard dual-cooled gel electrophoresis units (GE Healthcare, USA). The gels were run at 300 V for 3–4 h to separate small RNAs until the bromophenol blue dye reached the end of the gel, using a miRNA marker (NEB, cat. N2102S) as control. After that, the gel was stained with ethidium bromide, imaged on an AlphaImager 2200 (Alpha, USA) with the AlphaEaseFC software, and the amount and distribution of the RNAs were recorded.

### Transfer of Small RNAs to Nylon Membrane by ECL Semi-dry Blotter

For transfer of small RNAs, gels were placed over a sheet of nylon hybridization membrane (Hybond-NX, GE Healthcare) that had been pre-wetted in distilled water. This was then sandwiched between pieces of 3 MM Whatman filter paper (3–4 layers on each side), pre-wetted in 1×TBE buffer and placed in a TE77 PWR semi-dry transfer blotter (GE Healthcare) in 1×TBE buffer for 1 h at 400 mA.

### EDC Cross-linking of RNA to Membrane

The membrane, where the RNAs had been transferred, was placed on the EDC-saturated 3 MM paper, then wrapped in SARAN wrap, and incubated at 60°C for 2 h to facilitate RNA-membrane cross-linking [38]. The membrane was washed in distilled water to remove residual EDC solution prior to pre-hybridization, and was stored at −20°C until used.

### Hybridization, Washing and Exposure of the Membranes

After the EDC cross-link, the membrane was pre-hybridized with 20 ml hybridization buffer for 1–2 h at 42°C, followed by adding the labeled probes, and incubated at 42°C overnight for hybridization. In this research, antisense DNA oligonucleotides were end-labeled as probes with [γ-^32^P] ATP using T4 polynucleotide kinase (NEB), respectively (Table 1). Following the hybridization, the membrane was washed twice with washing buffer (2×SSC, 0.2% SDS) for 10–15 min at 42°C, and then exposed to a storage phosphor screen at room temperature overnight. Finally, the phosphor screen was scanned by Typoon TRIO variable mobile imager (GE Healthcare, USA).

**Table 1:** The primer and probe sequences used for detecting specific miRNAs and other small RNAs.

RNA	Primer/Probe	Length	Primer/Probe Sequence (5′-3′)*
miR159a	RT primer	50 nt	GTTGGCTCTGGTGCAGGGTCCGAGGTATTCGCACCAGAGCCAACctcctc
	Forward primer	21 nt	CGGCGGtttggattgaaggga
	Reverse primer	16 nt	GTGCAGGGTCCGAGGT
	Probe	21 nt	CAGAGCTCCCTTCAATCCAAA
U6 snRNA	Probe	25 nt	CTCGATTTATGCGTGTCATCCTTGC
miR403	Probe	21 nt	CGAGTTTGTGCGTGAATCTAG
miR162a	Probe	21 nt	CTGGATGCAGAGGTTTATCGA
tasiARFs (tandem)	Probe	42 nt	TGGGTCTTACAAGGTCAAGAAAAGGTCTTGCAAGGTCAAGAA
tasiR2141 (single)	Probe	21 nt	TGGGTCTTACAAGGTCAAGAA
miR390	Probe	21 nt	GGCGCTATCCCTCCTGAGCTT

*The lowercase letters indicate the reverse complementary nucleotides in the primer sequence. The specificity of stem-loop RT primers to individual miRNA is conferred by a 6-nt extension at the 3′ end; this extension is a reverse complement of the last six nucleotides at the 3′ end of the miRNA. Forward primers are specific to the miRNA sequence but exclude the last six nucleotides at the 3′ end of the miRNA.

### Detection of Mature miR159a by End-point Stem-loop RT-PCR

To test the feasibility of the small RNA isolation protocol, miR159a was amplified by the end-point stem-loop RT-PCR method [39]–[41]. A stem-loop containing RT primer with its 5′end complementary to the last 6 nt at 3′end of target miRNA was designed (the lower-case sequence was indicated) (Table 1). Reverse transcription was performed using 10 µg of small RNAs and 1 µM RT primer for miR159a. To remove RNA secondary structure, the mixture was first incubated at 65°C for 5 min and then chilled on ice for at least 2 min. Reverse transcription was performed at 16°C for 30 minutes, followed by 60 cycles of pulsed RT at 30°C for 30 s, 42°C for 30 s and 50°C for 1 s according to the manual of Reverse Transcription System Kit (Promega, USA). Finally, the reverse transcriptase was inactivated by incubation for 15 min at 70°C. PCR was performed using a forward primer containing the 5′terminal sequence of miRNA (the lower-case letters were indicated) and a universal primer complementing to the stem-loop part of RT primer. PCR was amplified at 94°C for 2 min, followed by 35 cycles at 94°C for 15 s and 60°C for 1 min [39]. The reaction products (61 bp) were analyzed by electrophoresis on a 4% agarose gel and then staining with ethidium bromide.

## Results

### Comparison with other Published Methods for Small RNAs Extraction

Several protocols, including CTAB protocol (CTAB extraction and LiCl precipitation) [21]–[23] and the LiCl protocol (LiCl extraction and LiCl precipitation) [25], have been reported to isolate small RNAs from various plant species. In addition, PEG8000 and LiCl are commonly used for the differential precipitation of HMW RNAs.

Prior to developing the new protocol, papaya leaves were used as a reference to evaluate the quality of isolated small RNAs according to the previously published protocols. Two different extraction buffers combined with several RNA precipitation reagents were tested for isolation and detection of miR159a, respectively.

**Figure 1:**
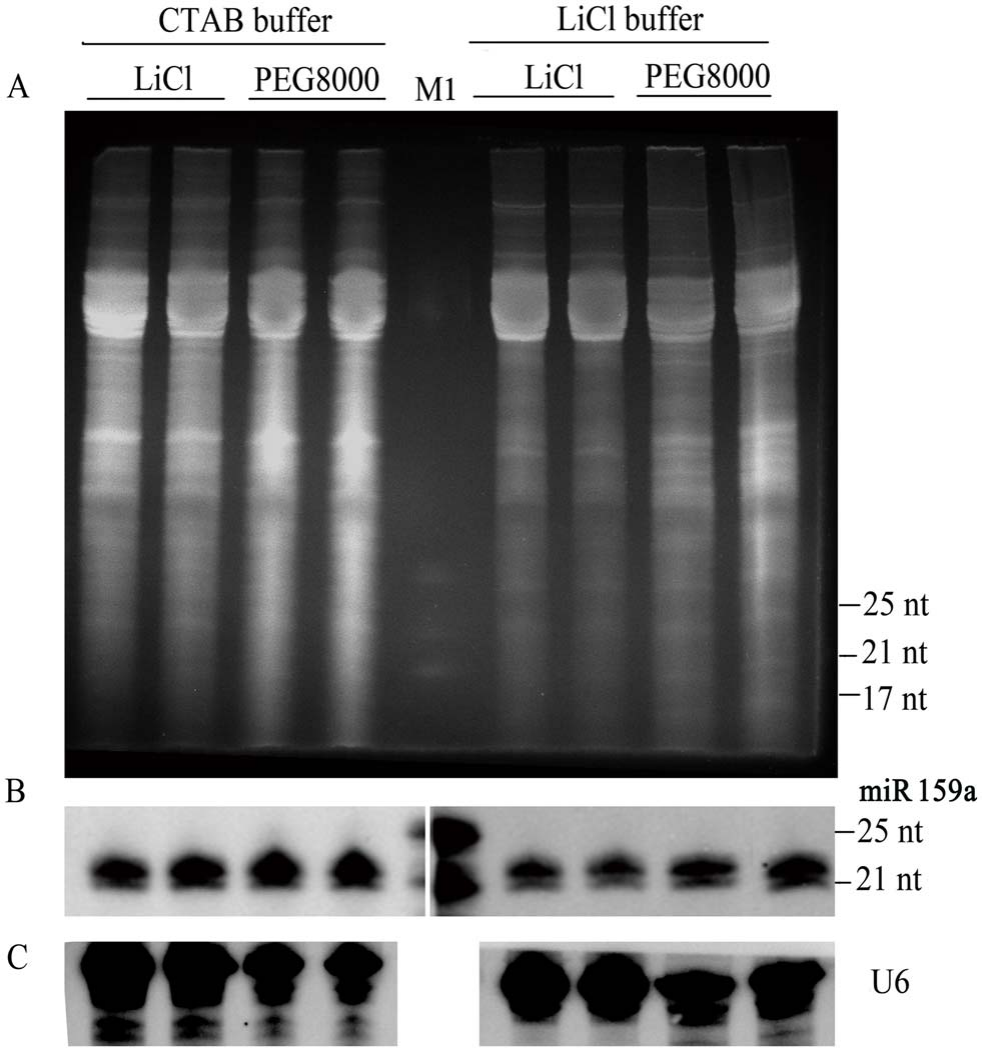
Comparison of small RNA extraction methods using papaya leaves. (A) Small RNAs extracted by different extraction buffers and separated from total RNA samples with various precipitation reagents. 60 µg of LWM RNAs extracted by different extraction methods were run on 17% polyacrylamide gel containing 7 M urea (20 volts/cm, 4 h), and visualized with ethidium bromide (EtBr) staining. (B) Northern blot assay of miR159a accumulation, and (C) U6 snRNA hybridization was used as a loading control. Lanes 1–2, identical sample, treated with CTAB extraction and LiCl precipitation; lanes 3–4, identical sample, treated with CTAB extraction and PEG8000 precipitation; lane 5, M1: miRNA marker (NEB), which is a set of three synthetic single-stranded RNA oligonucleotides of 17, 21 and 25 residues, respectively; lanes 6–7, identical sample, treated with LiCl extraction and LiCl precipitation; lanes 8–9, identical sample, treated with LiCl extraction and PEG8000 precipitation.

The results of gel electrophoresis showed that smear appeared in all lanes of loaded small RNA samples (Fig. 1A). The subsequent hybridization blots of miR159a showed two discrete bands on nylon membrane. Moreover, the main hybridization bands appeared above the band of 21-nt of the corresponding miRNA marker (Fig. 1B). According to previous studies [23]–[25], the main hybridization band of miR159a should appear at 21-nt, indicating that those extraction methods previously reported could hardly obtain high-quality small RNAs from papaya leaves. We speculate that the previously established protocols could not effectively eliminate the interfering substances, which might bind to RNA and form gelatinous complexes, thus affected the electrophoretic mobility of isolated small RNAs and resulted in smearing on the gels.

**Figure 2:**
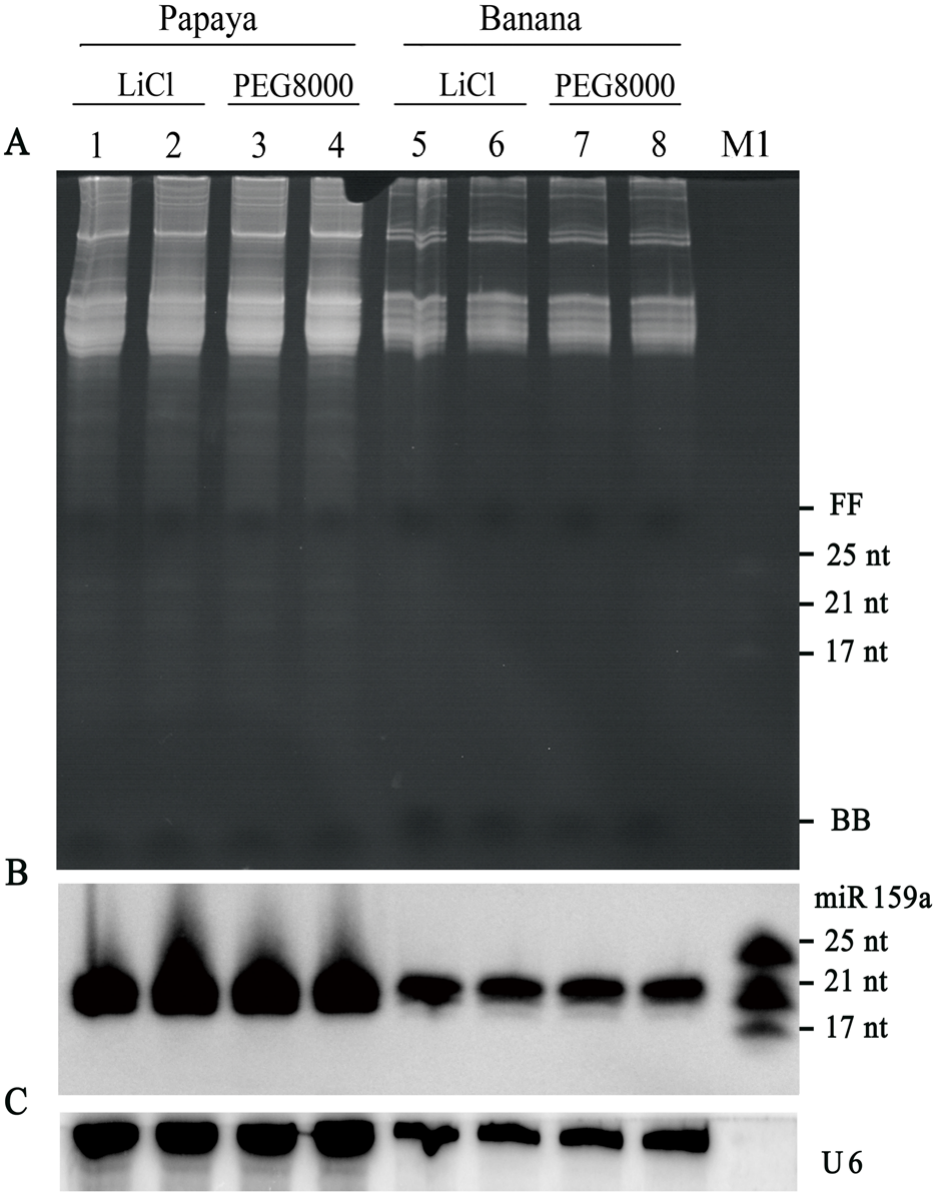
Analysis of small RNAs isolated from papaya and banana by the modified Trizol protocol with different precipitation reagents. (A) Gel electrophoresis of LMW RNAs from papaya and banana obtained with different precipitation reagents by modified Trizol protocol. A total of 60 µg of LWM RNAs were run on 17% polyacrylamide gels, and visualized with ethidium bromide (EtBr) staining. (B) Northern blot assay of miR159a accumulation, respectively, and (C) U6 snRNA hybridization was used as a loading control. Lanes 1–2 and 5–6 represent samples removing HMW RNA and DNA by PEG8000 in low salt concentration condition; Lanes 3–4 and 7–8 represent samples with selective precipitation of HMW RNA by 4 M LiCl. FF: xylene cyanol FF; BB: bromophenol blue; M1: miRNA marker.

In contrast, with the newly established small RNA extraction method, high-quality small RNAs were successfully isolated from both papaya and banana leaves. The clear background and distinct band of small RNAs were present on the gel (Fig. 2A). Furthermore, the miR159a blots showed a distinct and clear 21-nt (hybridization) band on the nylon membrane by either PEG8000 or LiCl precipitation reagent (Fig. 2B), confirming that the precursory step (step 1) could efficiently eliminate the interfering secondary metabolites and yield high-quality small RNAs.

Although either LiCl or PEG8000 reagent could result in high-quality small RNAs, we chose PEG8000 for subsequent HMW RNA precipitation in the extraction procedures, as the differential precipitation of HMW RNA with LiCl was rather time-consuming [42]. Furthermore, PEG was found to be more effective in removing polyphenolic compounds and polysaccharides [43].

### Isolated Small RNAs were Suitable for Detection of miRNAs and tasiRNAs

To date, there is only one experimentally validated miRNA precursor sequence (i.e. cpa-miR162a) available in miRNA Registry Database (miRBase version 19.0, http://microrna.sanger.ac.uk/) for papaya [44], [45]. It is known that miR162a expresses at low levels in *Arabidopsis* and rice, targeting DCL1, the main enzyme that processes pre-miRNAs into mature miRNAs in plants [46]–[48]. Additionally, another putative microRNA (i.e. cpa-miR403) predicted based on sequence homology was registered in PMRD database (http://bioinformatics.cau.edu.cn/PMRD/) [49], [50], which leads the cleavage of *AGO2* mRNA in *Arabidopsis* [7].

**Figure 3:**
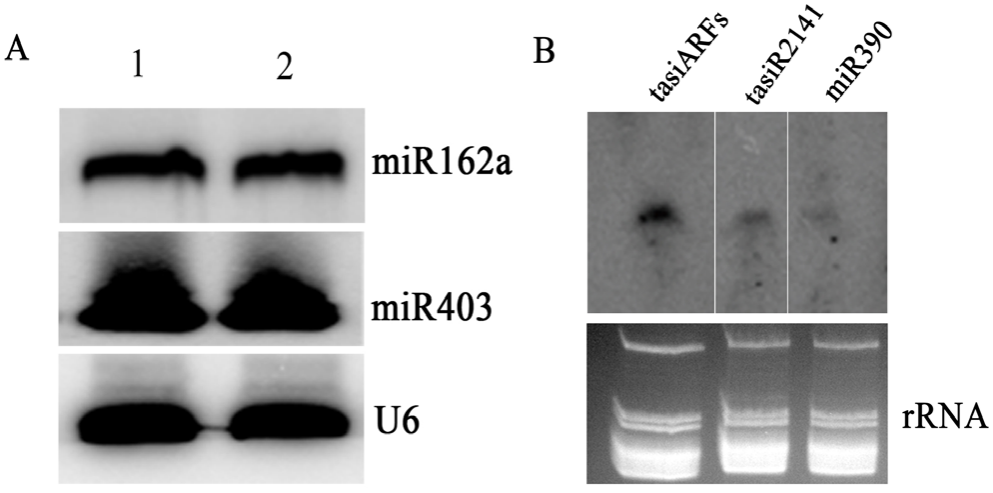
Experimental validation of papaya microRNAs by Northern blot assays. (A) Northern blot assays of papaya miR403 and miR162a each using 100 µg of LMW RNAs. U6 snRNA hybridization was used as a loading control. (B) Northern blot assays of papaya tasiARFs, tasiR2141 and miR390 based on sequence homology using 100 µg of LMW RNAs. Ethidium bromide-stained rRNAs are shown as a loading control.

To examine whether the small RNAs isolated using this newly established protocol were suitable for verifying the predicted miRNAs in papaya, small RNAs (100 µg) isolated from papaya leaves were used for the experimental validation of miR162a and miR403, respectively. The hybridization blot of miR403 showed cone-shaped band, while the blot of miR162a presented a clear and distinguishable band using the corresponding complementary oligonucleotide DNA probes, respectively, indicating that the two miRNAs were indeed present in papaya plants (Fig. 3A).

Besides miRNAs, we wonder whether the isolated small RNAs can be used for low-abundant siRNAs northern blotting analysis, especially conserved tasiARFs. The small RNAs (100 µg) isolated from papaya leaves were also used to detect putative conserved tasiARFs by northern blotting analysis. Both single (tasiR2141) or putative tandem tasiARFs (tasiR2141 and tasiR2142) that involved in *TAS3* biogenesis pathway were experimentally tested by northern blotting assay using corresponding complementary oligonucleotide DNA probes, and unambiguous blotting signals on nylon membrane indicated that the isolated small RNAs could be used for siRNAs detection and conserved tasiARFs existed in higher plants in deed (Fig. 3B). However, the miR390 blot signal was weak, indicating that miR390 accumulated at a low abundance in young papaya leaves. The results further confirmed the feasibility of newly established small RNAs extraction method and that *TAS3* family is highly conserved in higher plants.

### Small RNAs Extracted from Several Tropical and Subtropical Recalcitrant Plant Species

**Figure 4:**
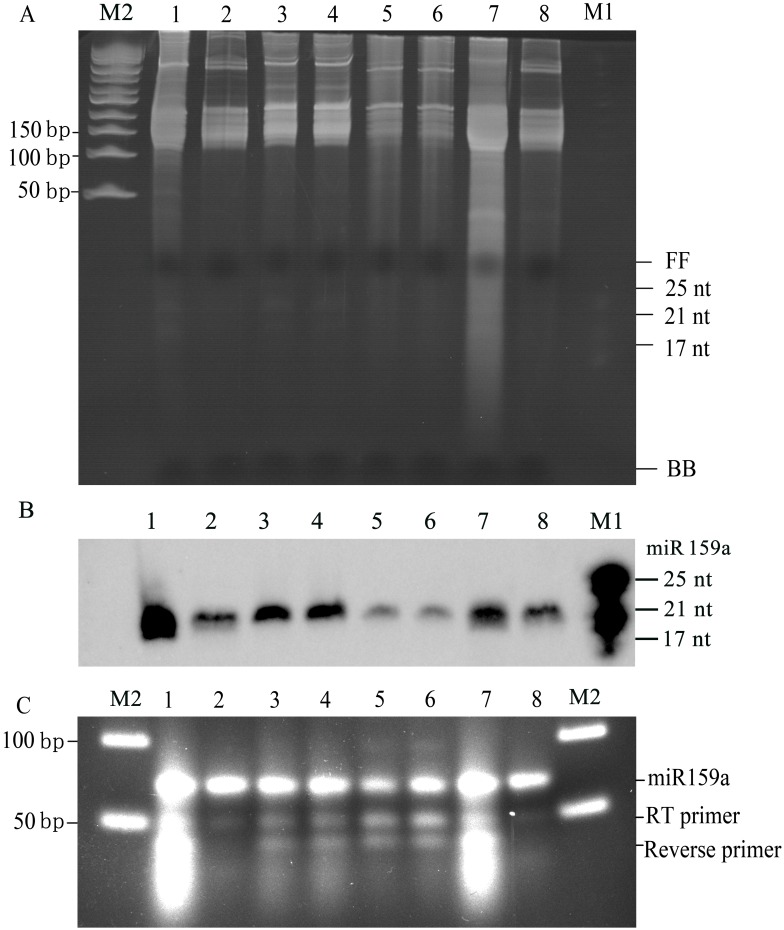
Analysis of small RNAs extracted from several tropical and subtropical plants. (A) Gel electrophoresis of LMW RNAs (60 µg) extracted from different tropical and subtropical plant leaves by the modified Trizol method. (B) Northern blot assay of miR159a accumulation, respectively. (C) End-point stem-loop RT-PCR (30 cycles) analysis showing the existence of mature miR159a. miR159a RT-PCR products, RT primer and forward primer used in end-point RT-PCR were visualized in the 4% agarose gel with ethidium bromide staining. Lanes 1–8 indicate guava, banana, citrus, pineapple, longan, lychee, rubber and cactus, respectively. FF: xylene cyanol FF; BB: bromophenol blue; M1: miRNA marker; M2: 50-bp DNA ladder.

We also attempted to isolate small RNAs from several other subtropical and tropical recalcitrant plant species as shown by the electrophoresis profile. Distinct bands, clear background and no smearing in all samples on the electrophoresis profiles showed that the small RNAs extracted from these plant species are of high purity (Fig. 4A). Distinct and sharp signals were obtained when the miR159a probe was hybridized with these isolated small RNAs (Fig. 4B). Furthermore, mature miR159a was also verified using an end-point stem-loop RT-PCR method. The reaction products (61 bp), RT primer (50 nt) and forward primer (21 nt) were also clearly visualized on 4% agarose gel with ethidium bromide staining (Fig. 4C).

**Figure 5:**
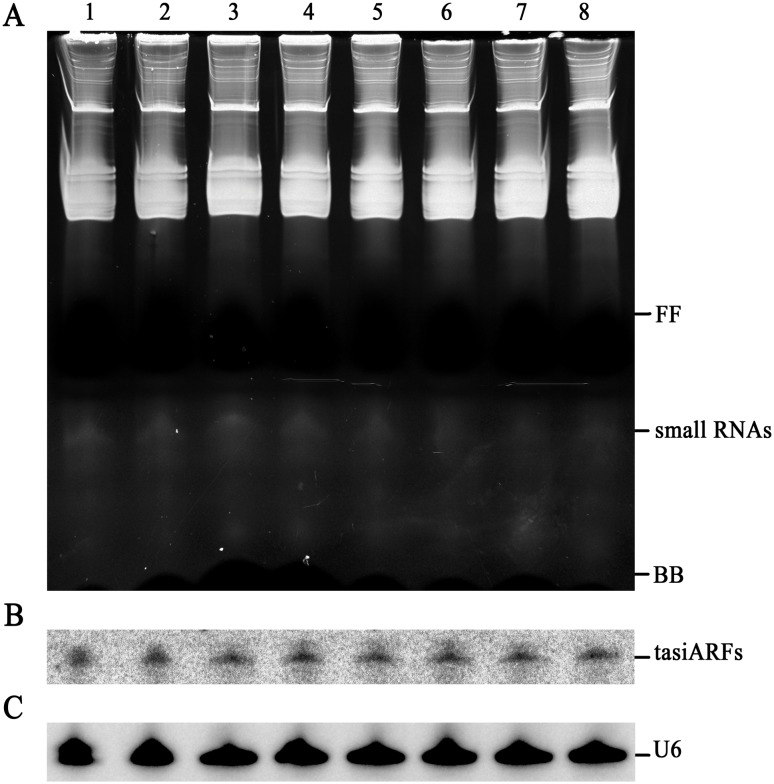
Analysis of putative conserved tasiARFs extracted from several tropical and subtropical plants. (A) Gel electrophoresis of LMW RNAs (100 µg) from different tropical and subtropical plant leaves. (B) Northern blot assay of putative conserved tasiARFs with a complementary 42-nt probe. (C) U6 snRNA hybridization is shown as a loading control. Lanes 1–8 represent guava, banana, citrus, pineapple, longan, lychee, rubber and cactus, respectively. FF: band of xylene cyanol FF; BB: band of bromophenol blue.

Furthermore, northern blotting was carried out to determine the expression of conserved tasiARFs, which has been known as of low abundance and further verified the availability of the small RNAs protocol described here. Distinct electrophoresis profile and clear background were visualized on the electrophoresis gel stained with ethidium bromide (Fig. 5A). Moreover, 100 µg of small RNAs isolated from these plant leaves were used for hybridization with the complementary 42-nt tandem putative tasiARF probe, and a well-defined band pattern was shown in Fig. 5B. The results showed that this small RNAs extraction method could be applied in a wide range of plant species, especially for recalcitrant plant species rich in secondary metabolites.

## Discussion

Extraction of high-quality small RNAs is an important procedure and can be a limiting factor in some experiments, such as northern blotting assay, and the investigation of specific miRNAs or siRNAs expression profiles using end-point RT-PCR amplification or microarray. The small RNAs isolation from recalcitrant plant species, especially from subtropical and tropical plant tissues, may be particularly difficult because of the presence of large quantities of polysaccharides and polyphenols and other substances that would co-precipitate with final resulted small RNAs and constitute the major obstacle of small RNAs isolation.

Several methods have been reported for the extraction of low-molecular-weight RNAs from different plants. However, these procedures were only used for model plants and could not recover small RNAs from recalcitrant plant tissues. Some of the available small RNAs isolation protocols were specially used for polysaccharide-rich materials, including LiCl extraction buffer and PEG8000 precipitation. However, theses protocols yield small RNAs of poor quality and low quantity from papaya leaves used in this study. Furthermore, commercially available small RNA extraction kits were not designed for use with plant tissues containing high concentrations of polyphenols and polysaccharides, and other secondary metabolites. To address this problem, we developed a new protocol for small RNAs extraction by modifying previously reported total RNA extraction buffer and combining current small RNAs isolation strategy to make it effective and compatible for a variety of recalcitrant plant tissues.

CTAB-based methodology has been successfully used for total RNA extraction from various plant tissues, which necessitated us to optimize the components in small RNAs extraction buffer and steps. In the extraction procedures, we also considered factors or reagents which facilitate the removal of polyphenolic compounds, precipitation of polysaccharides, and ribonuclease inhibition. CTAB, as a strong ionic denaturing detergent, has been proved useful in precipitating polysaccharides and widely used for both DNA and RNA extraction from plant tissues, especially various recalcitrant members of Euphorbiaceae [33]. Additionally, the guanidinium thiocyanate was added to inhibit RNase activity and prevent RNA degradation. Furthermore, PVPP instead of PVP was preferred because soluble PVP is incompatible with subsequent phenol extraction, which binds to nucleic acid and hinders RNA precipitation at the precursory step. PVPP is a strong H-receptor with high molecular weight and forms complexes with polyphenols effectively through hydrogen bonding, which could be separated from RNA by centrifugation [51]. Finally, high concentration of β-mercaptoethanol and EDTA were used for inhibiting the oxidization of polyphenol to polyquinones and the activities of enzymes as well [26], [52]. Moreover, higher concentration of NaCl could effectively separate the contaminating compounds in plant tissues [20]. In brief, the common interferences that prevented the isolation of high-quality small RNAs from recalcitrant plant tissues could be efficiently eliminated using CTAB-PVPP lysis buffer by a previous step of centrifugation before Trizol extraction. After the precursory step, total RNA was then extracted by Trizol, which is widely used for total RNA extraction in a time and cost effective way.

It was found that the resulting upper aqueous phase after chloroform treatment step co-incubated with an equal volume of PEG8000 (at 65°C for 15 min) was crucial for high-quality small RNAs isolation from recalcitrant plant tissues rich in polysaccharides and polyphenols. Since phenolic and other compounds that may interfere with RNA were bound to PEG8000 in extraction buffer, these remaining compounds were removed by centrifugation prior to small RNA recovery. Moreover, HMW RNA could be effectively co-precipitate with PEG8000 after centrifugation, which facilitated the recovery of high quality small RNAs.

The success of the RNA isolation protocol can be judged by the quality, quantity and integrity of small RNAs recovered. The high quality of small RNAs described here was confirmed in several ways. The *A260/A280* absorbance ratio was ranged from 1.7–2.2, and *A260/A230* absorbance ratio was 3.0–4.0 in average, indicating that small RNAs were relatively free of protein and substances. Second, 17% PAGE electrophoresis showed clear, discrete ribosomal RNA with apparent RNA degradation, suggesting that small RNAs was also relatively free of RNase. Third, the quality of small RNAs was tested by both northern blotting with miR159a and end-point RT-PCR, respectively. The results showed that high-quality small RNAs were obtained from different plant species by this modified method judged from both northern blotting and end-point stem-loop RT-PCR analysis. In addition, distinct and well defined bands were detected when using the putative tasiARF probes, ensuring that the purity of these small RNAs were sufficient for small RNA hybridization. To avoid the cross-contamination of samples between adjacent lanes, a 100-µl microsyringe with a long and thin needle was used for sample loading and the needle was pushed near the bottom of each well to prevent overflowing of samples.

Furthermore, the small RNAs prepared using the present protocol has been used for several other further investigations. Deep sequencing of the cold-responsive small RNAs in rubber tree (*Hevea brasiliensis*) and Fusarium wilt infection-associated small RNAs in banana (*Musa* spp.) have been finished using this protocol described here. In conclusion, the method reported here is useful for small RNA isolation from plants rich in polysaccharides, polyphenols and other secondary metabolites.
